# Effects of Grain Boundary Misorientation on the High-Cycle Fatigue Behavior of Nickel-Based Superalloy Bicrystals

**DOI:** 10.3390/ma19132735

**Published:** 2026-06-26

**Authors:** Qinghui Wu, Chenglu Zou, Xiuge Ma, Jianchao Pang, Zengqian Liu, Kailun Luo, Zhefeng Zhang

**Affiliations:** 1 Science and Technology on Advanced High Temperature Structural Materials Laboratory, AECC Beijing Institute of Aeronautical Materials, Beijing 100095, China; 2 Shenyang National Laboratory for Materials Science, Institute of Metal Research, Chinese Academy of Sciences, 72 Wenhua Road, Shenyang 110016, China

**Keywords:** bicrystal superalloy, misorientation, high-cycle fatigue, damage mechanism, fatigue life prediction model

## Abstract

Nickel-based single-crystal superalloys are key materials for manufacturing aero-engine turbine blades. Different grain boundaries are inevitably formed during the production of superalloys and weaken the fatigue performance of the alloys. Systematic exploration of the effect of grain boundary misorientation (GBM) on the fatigue properties of superalloys is of great significance. Available research cannot fully illustrate the influence of GBM on the high-cycle fatigue (HCF) damage mechanism of superalloys, especially its coupling with inherent casting defects. In this study, bicrystal specimens with misorientations of 4°, 8° and 12° were fabricated from a second-generation nickel-based single-crystal superalloy. The influence mechanism of misorientation variation on HCF performance was systematically investigated. The test results show that the HCF life of the alloy decreases obviously as GBM rises from 4° to 8° and then declines slowly. Fracture analysis indicates that fatigue damage is closely associated with GBM and casting defects. A 4° grain boundary promotes coordinated deformation and inhibits cracking, whereas misorientations over 8° cause dislocation pile-up and speed up crack propagation. Based on the significant effects of GBM and casting defects on fatigue damage behavior, as well as the analysis of the two key parameters in the Basquin model, a linear correlation is established between the fatigue strength coefficient (*σ*′_f_) and misorientation; a coupling relationship is constructed among the fatigue strength exponent (*b*), misorientation, and defect size. Prediction results confirm that the model achieves higher accuracy by incorporating casting defect parameters.

## 1. Introduction

In the manufacturing process of aeroengines, nickel-based superalloys, with their excellent high-temperature strength, oxidation resistance, and hot corrosion resistance, serve as the core material for critical components such as combustion chambers and turbine blades, directly determining the performance, service life, and reliability of the engine [1,2,3,4]. As representative new-generation primary materials for turbine blades of aeroengines, nickel-based single-crystal (SX) superalloys have a close correlation between their fatigue performance under complex alternating loads and the service life cycle and operational safety of the engine [5]. However, due to the limitations of the preparation process and the complexity of the internal cavity structure of turbine blades, intragranular defects such as subgrain boundaries, low-angle grain boundaries, or high-angle grain boundaries are inevitably introduced during the preparation of DD6, DD5, and other single crystals [6,7,8]. These grain boundaries compromise the integrity of the single-crystal structure, aggravate dislocation pile-up and stress concentration [9]. Meanwhile, such boundaries act synergistically with inherent casting defects to accelerate the initiation and propagation of fatigue cracks, thereby degrading the high-cycle fatigue (HCF) performance of single-crystal superalloys [10]. In engineering practice, it is necessary to define the grain boundary tolerance for single-crystal superalloys. When the grain boundary angle in the alloy exceeds a specific value, the alloy must be classified as a non-single-crystal structure, and its application must be restricted. Therefore, in-depth research on the influence of misorientation on the mechanisms of fatigue crack initiation and propagation in nickel-based superalloys can not only reveal the intrinsic correlation between crystal orientation and fatigue performance, but also provide important theoretical support for the precise design, process control, and life prediction of high-performance aeroengine hot-end components.

Grain boundaries are transition boundaries between grains with different crystal structure orientations, while grain boundary misorientation (GBM) mainly describes the degree of difference in crystallographic orientations between adjacent grains. It is generally believed that as the GBM increases, the grain boundary structure of superalloys gradually grows more complex, with precipitates starting to form at the grain boundaries [11]. In addition, rising misorientation leads to a gradual widening of the grain boundary, which reduces the bonding degree between grains on both sides [12]. When the misorientation reaches a sufficiently high level, white elongated precipitates begin to emerge at the grain boundaries, and their density rises remarkably with a further increase in misorientation [13,14]. For superalloys, grain boundary precipitates are harmful phases; an increase in precipitates will gradually degrade the performance of superalloys [15]. Hu et al. [16] reported that rising misorientation elevates grain boundary interfacial energy and promotes abundant lamellar M_23_C_6_ precipitation, deteriorating grain boundary cohesion and alloy strength. During fatigue damage evolution, grain boundaries become the key location for fatigue crack initiation controlled by crystallographic slip. Miao et al. [17] clarified that concentrated plastic slip activated on {111} slip planes adjacent to grain boundaries serves as the primary origin of fatigue crack nucleation; uneven slip accumulation near grain boundaries causes local stress concentration, whereas the capability of slip transfer across different grain boundaries strongly determines the propagation route of microstructurally short fatigue cracks [18]. It is thus clear that variations in grain boundaries and their misorientation significantly alter both the static mechanical properties and fatigue performance of superalloys.

In addition, affected by casting processes, casting defects such as shrinkage pores and micropores are inevitably generated during the preparation of superalloys, and such defects significantly regulate the fatigue performance of the alloys [19]. Study [20] has shown that under cyclic loading, micropores and non-metallic inclusions tend to couple with adjacent grain boundaries, acting as preferential initiation sites for fatigue cracks and thereby deteriorating the HCF performance of the alloy. From the perspective of thermodynamic entropy production theory, Ding et al. [21] further revealed that the synergistic damage between casting defects and grain boundary precipitates remarkably increases the fatigue entropy production rate; after reducing the fatigue fracture entropy by nearly 30% through grain boundary modification treatment, the fatigue performance of the material can be significantly optimized. Notably, defect size is one of the core factors affecting the fatigue life of the alloy [22]. By designing controlled experiments with defects of different sizes, Kevinsanny et al. [23] confirmed that the matching relationship between defect size and grain boundary microstructure directly determines the initiation location and propagation path of fatigue cracks. It is evident that the influences of casting defects and grain boundary characteristics on the fatigue performance of superalloys have been widely verified. Nevertheless, few quantitative studies have simultaneously considered GBM, casting defect size and HCF life for bicrystalline specimens of nickel-based single-crystal superalloy, and the quantitative coupling relationship among the three factors has not been fully developed so far. Therefore, further systematic research is still needed to quantitatively measure the extent of their impacts and ultimately establish a quantitative fatigue life prediction model with high prediction accuracy.

In this study, the HCF performance of typical nickel-based bicrystal superalloys with grain boundary misorientations of 4°, 8° and 12° is characterized, and the underlying mechanism for the effect of misorientation on fatigue damage is systematically explored. Combined with the revealed damage mechanisms, an innovative fatigue life prediction model is developed.

## 2. Material and Experimental Procedure

**Figure 1:**
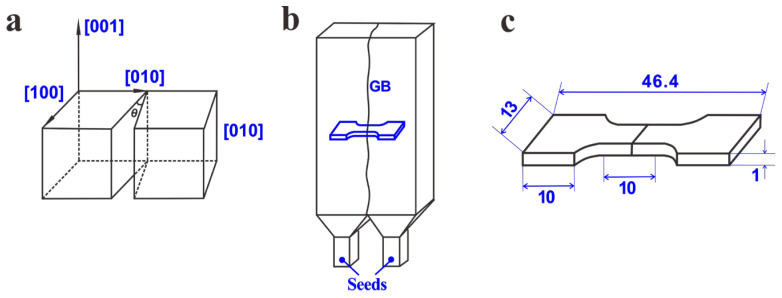
Schematic diagram of the two-crystal-seed method and fatigue sample: (**a**) orientations of seed crystals [24]; (**b**) cutting position of sample; (**c**) shape and dimension of fatigue sample (mm).

A second-generation nickel-based SX superalloy was used as the starting material in this study. The chemical composition was analyzed using a plasma emission spectrometer (iCAPQ, Thermo Fisher Scientific, Waltham, MA, USA), and the results are presented in Table 1. Bicrystal plates were prepared via the double seed crystal method combined with directional solidification technology, and the entire process was carried out under vacuum conditions at a melting temperature of 1580 °C. Two identical seed crystals were placed at both ends of the bottom of the mold shell, with both seed crystals deviating from the [001] orientation by a specific angle, as shown in Figure 1a. One of the seed crystals was rotated by an angle *θ* relative to the other around the [001] orientation as the rotation axis. Subsequently, the high-temperature molten metal was gradually poured into the mold shell of a fixed shape, and the columnar crystals were solidified and grown along the [001] direction by controlling the temperature gradient, resulting in the simultaneous growth of the two seed crystals into single crystals.

During the growth process, the two single crystals with different orientations bond with each other, eventually forming a bicrystal plate containing a tilt grain boundary with a specific angle, as schematically shown in Figure 1b. The GBM depends on the relative deviation angle *θ* between the seed crystals, and the grain boundary type is determined by the orientation relationship between the seed crystal rotation axis and the grain boundary plane. During the preparation process, the [001] rotation axis is parallel to the grain boundary plane, so the bicrystal is a tilt grain boundary bicrystal.

Bicrystal plates of nickel-based superalloy with grain boundaries of specific misorientations (4°, 8°, and 12°) were fabricated for systematic experimental research. The as-cast bicrystal plates were subjected to standard heat treatment with the schedule: 1290 °C/1 h + 1300 °C/2 h + 1315 °C/4 h/AC + 1120 °C/4 h/AC + 870 °C/32 h/AC. The schematic diagram of fatigue sample cutting is shown in Figure 1b, where the grain boundary is located in the middle of the parallel section of the sample, and the specific dimensions of the sample are presented in Figure 1c. The grain boundary is perpendicular to the stress loading direction, so the grain boundary plane bears the maximum normal stress. After machining, the surfaces were ground stepwise using SiC abrasive papers from 1200# to 2000#, followed by mechanical polishing with 2.5 μm diamond suspensions. A GPS-20 high-frequency fatigue testing machine was used for the HCF tests, with a tension–tension fatigue loading mode and a stress ratio R = 0.1. The test frequency was approximately 100 Hz, the loading waveform was sinusoidal, and the test temperature was room temperature. Two valid fatigue data points were obtained for each specified stress level and GBM condition. Specimens were defined as run-out when the fatigue life achieved 10^7^ cycles. The metallographic specimens and fracture morphologies were examined in secondary electron (SE) mode using a Hitachi JSM-6510 scanning electron microscope (SEM, Hitachi High-Tech, Tokyo, Japan) equipped with an energy dispersive X-ray spectrometer (EDS, Hitachi High-Tech, Tokyo, Japan) at room temperature. Prior to SEM observation, metallographic specimens were sequentially subjected to grinding, polishing and macro-etching; the macro-etchant consisted of 50 vol.% concentrated hydrochloric acid and 50 vol.% hydrogen peroxide, with specimens immersed in the mixed solution in a fume hood for 15 min, followed by ethanol cleaning and drying for subsequent grain boundary marking. The defect area was measured using image pro plus (IPP) software (Version 6.0, Media Cybernetics, Bethesda, MD, USA).

## 3. Results

### 3.1. Microstructure

**Figure 2:**
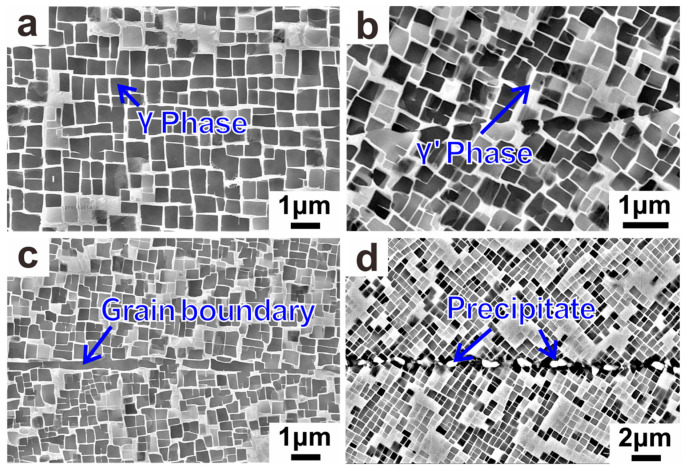
SEM (SE) micrographs of grain boundaries with different misorientations: (**a**) 0°; (**b**) 4°; (**c**) 8°; (**d**) 12°.

The matrix microstructure of the second-generation nickel-based SX superalloy is mainly composed of the γ matrix phase with lower strength and the γ′ precipitation strengthening phase with higher strength. Figure 2 presents the grain boundary microstructure of the heat-treated nickel-based superalloy bicrystals. This morphology is obtained by chemical etching, so the γ matrix phase appears white while the γ′ precipitation strengthening phase is black. The γ phase forms a grid-like network with uniform line width and homogeneous distribution, and the γ′ phase exists as square-shaped precipitates with similar sizes and straight edges. The two phases exhibit a brick wall-like distribution.

GBM has a significant influence on the grain boundary structure and the morphology of γ′ phases near the grain boundaries. For comparison with bicrystal specimens, the microstructure of the 0° misorientation sample (single crystal) is shown in Figure 2a. Its grain boundary is hardly distinguishable owing to high coherency between adjacent grains. The γ′ precipitates across the boundary remain continuous and intact without separation, showing uniform cubic morphology within the γ matrix. According to measurements, the overall average γ′ size is approximately 0.5 μm, the average width of γ matrix channels is 0.15 μm, and the average area fraction of γ′ phase is about 61%.

When the GBM is small (4°, Figure 2b), the grain boundary width is narrow, and the grains on both sides are closely bonded. Continuous white strip-like γ phases exist at the grain boundaries, indicating that the grain boundaries are composed of the γ matrix phase, with incomplete black γ′ phases cut by the grain boundaries on both sides. As the GBM increases (8°, Figure 2c), the grain boundary width increases significantly, indicating a decrease in the bonding capacity between grains on both sides of the grain boundaries. Meanwhile, continuous strip-like deformed black γ′ phases appear at the grain boundaries, connecting with the incomplete γ′ phases cut by the grain boundaries. With a further increase in GBM (12°, Figure 2d), the grain boundary width increases further, the deformed γ′ phases at the grain boundaries coarsen significantly, and continuous white precipitates are formed at the grain boundaries. EDS was performed on these white precipitates, and the measured compositional data are listed in Table 2. The EDS results reveal obvious enrichment of refractory elements such as W, Mo and Re, indicating that these precipitates are highly likely TCP phase.

### 3.2. Fatigue Property

**Figure 3:**
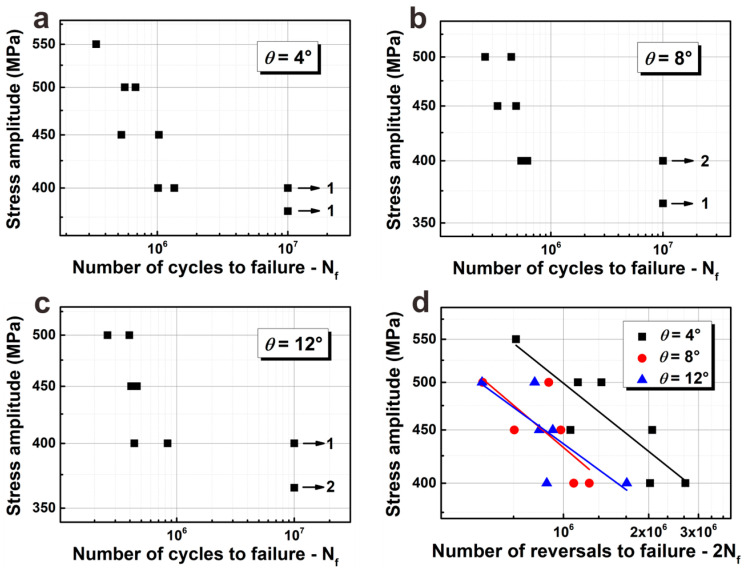
Fatigue life under different GBMs: (**a**) 4°; (**b**) 8°; (**c**) 12°; (**d**) Basquin model fitting of the fatigue data.

Figure 3 shows the HCF life of the nickel-based superalloy bicrystals under different GBMs. As shown in Figure 3a–c, the fatigue life increases significantly with decreasing stress amplitude for all misorientation conditions.

To compare the fatigue life variation under the three misorientations, the number of reversals to failure is used as the horizontal axis in Figure 3d, and the fatigue data are linearly fitted on a double-logarithmic scale based on the Basquin model. The results indicate that, at the same stress amplitude, the fatigue life of specimens with *θ* = 4° is significantly higher than those with *θ* = 8° and 12°. The fitted curves for *θ* = 8° and 12° are close to each other, showing little difference in their fatigue life variation trends. It can thus be concluded that the HCF life of the superalloy bicrystals generally declines as the GBM increases. The fatigue life decreases markedly when the misorientation rises from 4° to 8°, while the fatigue performance tends to change gently with a further increase in misorientation from 8° to 12°.

### 3.3. Fatigue Fracture Morphology

During cyclic loading, the deformation mode of superalloys is dominated by slip, whose driving force may be influenced by the coupling of multiple factors such as crystallography, microstructure, and alloy composition. To better understand the fatigue fracture process of the superalloy bicrystals with different misorientations, fatigue fracture surfaces were observed.

**Figure 4:**
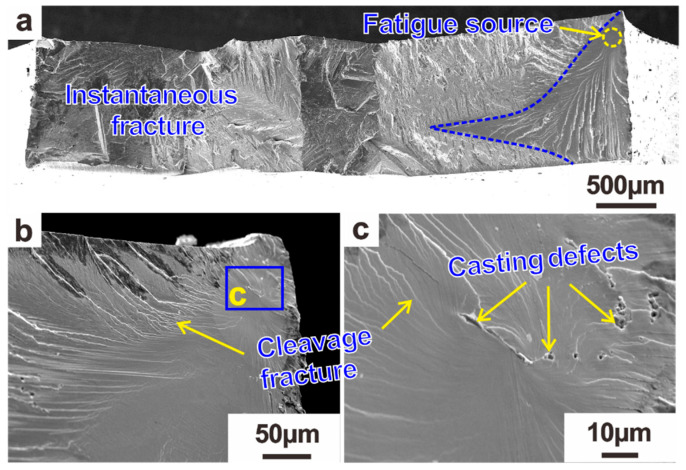
Fatigue fracture morphology of superalloy bicrystal with a misorientation of 4°: (**a**) overall fracture morphology; (**b**) fatigue crack initiation site morphology; (**c**) local magnified image of the blue square area.

Figure 4a shows the macroscopic fatigue fracture morphology at a misorientation of 4°. It can be observed that the fatigue crack initiation site is located in the subsurface of the sample. After crack initiation, the overall slow propagation zone presents an approximately triangular shape (as indicated by the blue dashed line in Figure 4a) instead of a typical fan-shaped radial zone, indicating that the crack propagation process may have encountered certain obstacles. In addition, a large number of clear radial patterns are present in the slow propagation zone, which is a typical characteristic of brittle fracture. Figure 4b and Figure 4c are images of the fatigue crack initiation site and its local magnified view, respectively. Obvious casting defects can still be observed near the fatigue crack initiation site (Figure 4c), indicating that the micropores formed during the casting process remain the main cause of fatigue crack initiation. After crack initiation, river-like cleavage patterns are rapidly formed in the matrix. The slip characteristics at 4° misorientation are not obvious, which may be due to the blocking effect of grain boundaries on dislocations.

**Figure 5:**
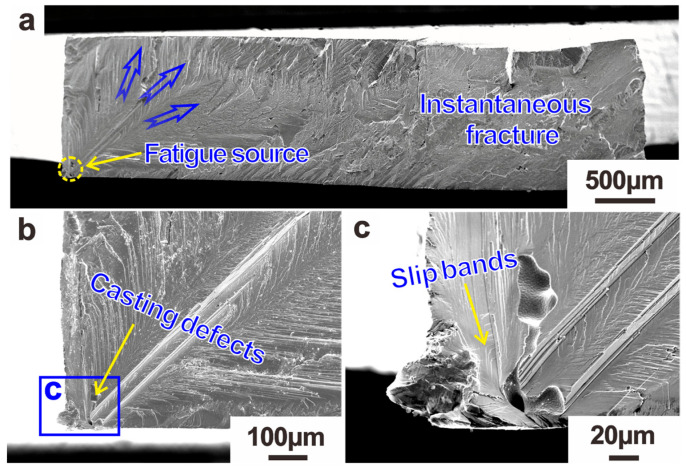
Fatigue fracture morphology of superalloy bicrystal with a misorientation of 8°: (**a**) overall fracture morphology; (**b**) fatigue crack initiation site morphology; (**c**) local magnified image of the blue square area.

Figure 5 shows the fatigue fracture morphology of superalloy bicrystal with a misorientation of 8°. It can be observed that the fatigue crack initiation site occurs at the vertex of the rectangular fracture (as shown in Figure 5a). After initiation, the fatigue crack grows radially in the direction of the blue arrow in Figure 5a, and a large number of parallel-arranged slip bands are clearly visible.

From the local magnified image of the fatigue crack initiation site in Figure 5b, it can be found that there are a large number of irregularly shaped casting pores in the initiation site. The stress concentration caused by these casting pores is considered to be the main reason for the initiation of fatigue cracks. After crack initiation, river-like fracture patterns are rapidly formed in the matrix, indicating that brittle fracture is still the dominant fracture mode at this time. Two obvious slip band-like structures extend from the fatigue crack initiation site (as shown in Figure 5b,c), which may be caused by the deformation incompatibility of the matrices on both sides of the bicrystal under a large misorientation.

**Figure 6:**
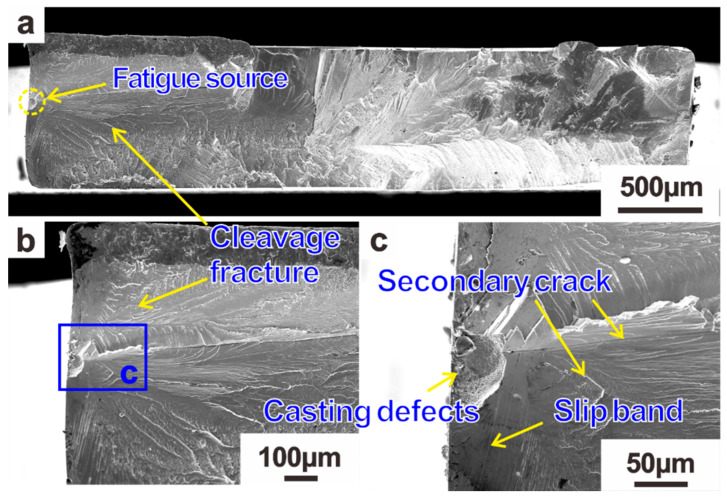
Fatigue fracture morphology of superalloy bicrystal with a misorientation of 12°: (**a**) overall fracture morphology; (**b**) fatigue crack initiation site morphology; (**c**) local magnified image of the blue square area.

Figure 6 shows the HCF fracture morphology of superalloy bicrystal with a misorientation of 12°. It can be observed that the fatigue crack initiation site still occurs at the edge of the sample (as shown in Figure 6a), and obvious circular casting defects are visible (as shown in Figure 6b,c). This indicates that even under a large misorientation, casting defects remain the main cause of fatigue crack initiation in this type of alloy. Due to the more significant crystallographic misorientation, a strong distorted stress field exists at the grain boundary. At this time, the superposition of stress concentration caused by casting defects leads to intense slip, resulting in very obvious signs of crack propagation. Obvious coarse slip bands and secondary cracks can be observed (as shown in Figure 6c).

## 4. Discussion

### 4.1. HCF Damage Mechanism

**Figure 7:**
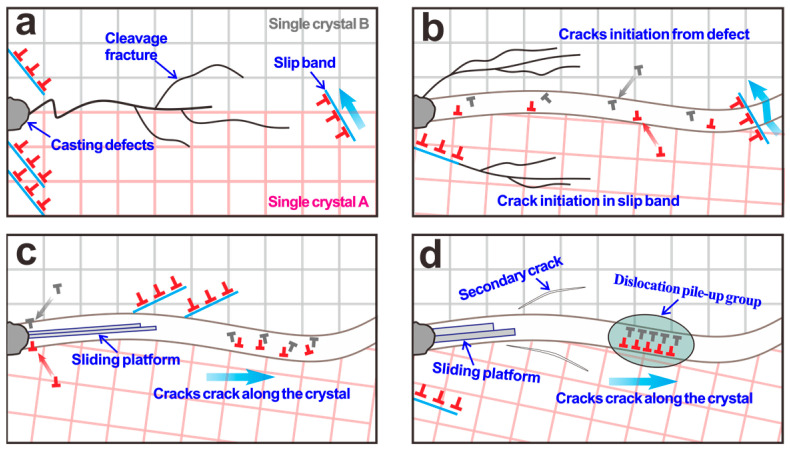
Schematic diagrams of the proposed speculative fatigue damage mechanism of superalloy bicrystals under different misorientations: (**a**) 0° single-crystal; (**b**) 4° bicrystal; (**c**) 8° bicrystal; (**d**) 12° bicrystal. (Red and gray represent two single crystals, however, they are actually one single crystal in (**a**)).

Misorientation modulates the HCF life of nickel-based superalloy bicrystals by altering grain boundary structures, dislocation motion modes, and the modes of fatigue crack initiation and propagation, with distinct damage mechanisms exhibited at the studied misorientations of 4°, 8°, and 12° for bicrystal samples. For comparative reference, the key deformation feature of the 0° single-crystal sample is briefly analyzed here (Figure 7a): since no grain boundary exists in the single-crystal structure, dislocations and slip bands may move freely inside the grain [12]. Such behavior may contribute to relatively uniform deformation and help avoid severe local stress concentration [11], while fatigue cracks may preferentially nucleate at obvious casting defects if these defects are present inside the specimen (Figure 7a). This provides a baseline for understanding the regulatory role of grain boundaries in the fatigue damage behavior of the bicrystal superalloys discussed below.

Figure 7b is a schematic diagram of the HCF damage mechanism of superalloy bicrystal at a misorientation of 4°. At this time, slight lattice deflection causes partial atomic rows to be unable to align completely, and misfit dislocations are formed to compensate for the orientation misfit at the grain boundary [12,25]. Nevertheless, the atomic arrangement still maintains high continuity, and the grain boundary is composed of a low-density dislocation network with a low grain boundary energy level [26,27]. This grain boundary state is likely to achieve a balance between the strengthening effect of the weak coherent grain boundary and the stress release capacity [28,29]. Most boundary atoms retain lattice matching, with sparse dislocations relieving minor mismatch. Coherency declines progressively with growing misorientation. In the early stage of cyclic loading, large-sized casting defects remain the main source of stress concentration (as shown in Figure 4c and Figure 7b). However, it is undeniable that the weak coherent grain boundary may promote the short-range movement of dislocations near the grain boundary along the grain boundary, thereby dispersing the stress at the defect tip and delaying crack initiation. With the progress of cyclic loading, cracks may initiate at two types of locations: one at casting defects, and the other at local concentrated regions of slip bands (as shown in Figure 4b,c and Figure 7b). The multi-site crack initiation makes the stress concentration at the defects dispersed, which increases the difficulty of rapid convergence of microcracks into a main crack in the early stage of crack propagation. This is one of the reasons why the fatigue life is significantly higher at a misorientation of 4°.

When the misorientation is further increased to 8°, the grain boundary coherency appears to be further lost, which may lead to a significant increase in the density of misfit dislocations near the grain boundary with disordered distribution and a marked decrease in dislocation mobility. Under cyclic loading, when dislocations in the grains slip to the grain boundary, they cannot pass through as smoothly as in the case of lower misorientations such as 4° [30]. Instead, they accumulate in large quantities at the grain boundary to form dislocation pile-ups (as shown in Figure 7c). At this time, the dislocation pile-up at the grain boundary and the stress concentration at the defects produce a synergistic effect. By intensifying local stress concentration and activating additional dislocation sources, it promotes the formation of slip bands over a wider range, ultimately leaving obvious step-like banded features and corresponding slip platforms in the fracture (as shown in Figure 5b and Figure 7c). It can be seen that with the increase in misorientation, the fatigue crack initiation process of superalloy bicrystal has gradually transformed from being defect-dominated to being co-dominated by defects and grain boundaries. The decreased continuity of atomic arrangement near the grain boundary accelerates the dislocation pile-up process, and it produces a synergistic effect with damage factors such as casting defects and slip bands, ultimately resulting in a significant reduction in fatigue life compared with that at a misorientation of 4°.

When the misorientation of superalloy bicrystal reaches 12°, it is close to the category of high-angle grain boundaries. During cyclic loading, dislocations gradually accumulate in large quantities at the grain boundaries with a disordered distribution. When dislocations moving along slip systems in the grains migrate to the grain boundaries, they may be hindered by the disordered dislocations and gradually form ultra-high density dislocation pile-ups. This may raise the local stress concentration factor and suggests an enhanced driving force for grain boundary crack initiation [31,32]. At this time, if there are large-sized casting defects near the grain boundaries, the stress concentration at the defects will superimpose with the stress from dislocation pile-ups at the grain boundaries. This further temporarily activates the matrix slip systems, forms local slip bands, and leaves obvious slip platforms in the fracture (as shown in Figure 6c and Figure 7d). The stress field during the propagation of the main crack will activate the surrounding weak regions and form secondary cracks. The secondary cracks quickly converge with the main crack through the stress field (Figure 6c and Figure 7d), significantly increasing the width of the main crack. This ultimately accelerates the intergranular unstable fracture of the sample. Notably, 12° and 8° specimens exhibit similar fatigue lives despite distinct grain boundary damage and crack growth. This mainly stems from grain-boundary damage saturation, as both quickly reach saturated damage via dense dislocation pile-ups under cyclic loading, suppressing intrinsic life differences.

### 4.2. Fatigue Life Prediction

Based on the damage mechanisms described earlier, the coupling law between misorientation and casting defects is clear. This coupling mechanism indicates that it is necessary to incorporate defect characteristic parameters and grain boundary damage characteristics related to misorientation into a unified prediction framework to improve the accuracy and reliability of life assessment.

In Figure 3d, the Basquin model was used to fit the relationship between stress amplitude and the number of reversals to failure for the superalloy bicrystals with different GBMs. The expression of the Basquin model is given as follows:

(1) $$\sigma_{a}={\sigma^{\prime }}_{f}\cdot {(2N_{f})}^{b}$$

where *σ*′_f_ is the fatigue strength coefficient; *b* is the fatigue strength exponent.

**Figure 8:**
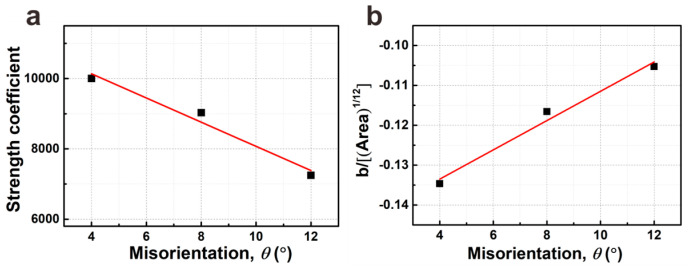
Prediction process of fatigue strength coefficient and fatigue strength exponent: (**a**) Linear relationship between fatigue strength coefficient and misorientation; (**b**) Relationship between fatigue strength exponent, defect size, and misorientation.

A large number of studies [33,34,35] have shown that the fatigue strength coefficient is usually positively correlated with the tensile strength of the material. In superalloy bicrystals with different misorientations, the tensile strength is mainly dominated by the misorientation and is relatively insensitive to general-sized defects under conventional testing conditions. Therefore, in this study, the fatigue strength coefficient is correlated with misorientation, and the results are shown in Figure 8a. It can be observed that the fatigue strength coefficient exhibits an obvious linear decreasing trend with the increase in misorientation, which is consistent with the variation trend of tensile strength with misorientation [36]. Thus, a quantitative relationship for the fatigue strength coefficient can be derived as follows:

(2) $${\sigma^{\prime }}_{f}=\alpha \cdot \theta +\beta$$

where *α* and *β* are fitting constants. The fitted values are *α* = −344 and *β* = 11,513.

As another key parameter in the Basquin model, the fatigue strength exponent typically characterizes the ability of cyclic load to convert into actual fatigue damage during cyclic loading [37,38]. A larger absolute value of b indicates a stronger ability to translate cyclic load into fatigue damage. Based on the previous analysis [12,39], in bicrystal superalloys, a larger misorientation leads to a higher atomic mismatch degree at the grain boundary, making it more prone to forming stress concentration and inducing fatigue crack initiation. Additionally, it can be observed from the fatigue fracture micrographs in Figure 4, Figure 5 and Figure 6 that defects also play a crucial role in fatigue crack initiation; larger defect sizes result in lower resistance to fatigue crack initiation. Evidently, the fatigue strength exponent is proportional to both the magnitude of misorientation and defect size.

Among the various discussions [40,41] on material defects and HCF properties, the viewpoint proposed by Murakami et al. [42,43] has been widely recognized in the relevant field. They argued that the HCF properties of metallic materials are usually inversely proportional to the projected area of inclusions or defects. When the area is less than 1000 μm^2^, Murakami suggested that the exponent *n* should be taken as 6 [43]. Therefore, in this study, ${\left(\sqrt{Area}\right)}^{1/6}$ is used to represent the defect size. Based on the previous analysis, it can be concluded that the fatigue strength exponent is closely related to both misorientation and defect size. Thus, a correlation is established between the ratio of the fatigue strength exponent to the defect size and the misorientation, with the results shown in Figure 8b. It can be observed that as the misorientation increases, the ratio of the fatigue strength exponent to the defect size also gradually increases, and the two exhibit an approximately linear relationship. Therefore, the following can be derived:

(3) $$\frac{b}{{\left(\sqrt{Area}\right)}^{1/6}}=\rho \cdot \theta +\omega$$

where *ρ* and *ω* are fitting constants. The fitted values are *ρ* = 0.0036 and *ω* = −0.148. It can be seen that reducing either the misorientation or the defect size can decrease the value of *b* to a certain extent, thereby reducing the ability to convert cyclic loading into actual fatigue damage to the material and further improving the fatigue resistance of the material.

Based on the above analysis, the two key parameters (*σ*′_f_ and *b*) in the Basquin model are equivalently substituted with misorientation and defect size, respectively. Therefore, the fatigue life can be further regarded as a function of stress amplitude, misorientation, and defect size, with its expression given by:

(4) $$\sigma_{a}=\left(\alpha \cdot \theta +\beta \right)\cdot {(2N_{f})}^{\left(\rho \cdot \theta +\omega \right){\left(\sqrt{Area}\right)}^{1/6}}$$

**Figure 9:**
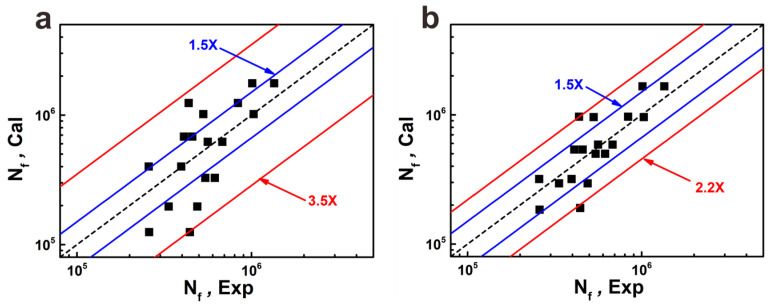
Fatigue life prediction results: (**a**) without incorporating the defect size parameter; (**b**) incorporating the defect size parameter.

Based on the above relationships, after determining the corresponding fitting parameters, the HCF life of bicrystal superalloys can be predicted, and the prediction results are shown in Figure 9. For comparison, the prediction results without incorporating defect parameters for modification are presented in Figure 9a. It can be observed that without considering the defect parameter, the prediction error of the model reaches as high as the 3.5× error band (see Figure 9a). By contrast, after introducing the defect parameter, the model error is reduced to the 2.2× error band. In addition, 78% of the predicted points fall within the 1.5× error band with the defect size parameter incorporated (see Figure 9b)., while this value is merely 44% without considering the defect size parameter. This fully demonstrates that the proposed fatigue life prediction model, by incorporating the defect size parameter, can effectively predict the HCF life of bicrystal superalloys at different misorientations. This model shows good adaptability to the materials used in the present work. Its general applicability to other materials awaits further exploration.

## 5. Conclusions

Based on the study of HCF performance, damage mechanisms, and fatigue life prediction of nickel-based superalloy bicrystals with different misorientations, the main conclusions are drawn as follows:

1. As GBM rises from 4° to 8°, the HCF life declines by nearly 50% under equivalent applied stress, while fatigue life remains nearly unchanged when misorientation increases from 8° to 12°.
2. Casting defects dominate fatigue crack initiation in the bicrystals. Increased misorientation promotes secondary cracking and accelerates crack propagation. Misorientation controls damage via grain boundary and dislocation behavior: low 4° misorientation boundaries mitigate local stress concentration to retard crack initiation, whereas deteriorated coherence at 8° and 12° boundaries triggers heavy dislocation pile-up to accelerate cracking.
3. By modifying the Basquin equation, a novel fatigue life prediction model is developed for bicrystal superalloys under the present tested fatigue conditions. The fatigue strength coefficient (*σ*′_f_) is linearly correlated with misorientation, and the fatigue strength exponent (*b*) is coupled with both misorientation and defect size. Validation results show that incorporating the defect parameter significantly improves prediction accuracy, reducing the error band from 3.5× to 2.2×.

## Figures and Tables

**Table 1:** Chemical compositions of the studied single crystal superalloy (wt. %).

	Cr	Al	Mo	W	Ta	Re	Nb	Hf	C	Ni
9.05	4.45	5.57	1.98	7.53	7.19	2.26	1.05	0.11	0.015	bal

**Table 2:** Chemical composition of precipitated phase measured by EDS.

Element (K)	W	Mo	Re	Fe	Co	Ni	Nb	Al	Ta	Cr	C
Weight (%)	30.88	11.42	5.85	0.24	5.88	17.43	1.64	0.81	8.63	5.29	11.92

## Data Availability

The original contributions presented in this study are included in the article. Further inquiries can be directed to the corresponding authors.
